# Concurrent enhancement of percolation and synchronization in adaptive networks

**DOI:** 10.1038/srep27111

**Published:** 2016-06-02

**Authors:** Young-Ho Eom, Stefano Boccaletti, Guido Caldarelli

**Affiliations:** 1 IMT School for Advanced Studies Lucca, Piazza San Francesco 19, 55100 Lucca, Italy; 2 Departamento de Matemáticas, Universidad Carlos III de Madrid, 28911 Leganés, Spain; 3 CNR-Istituto dei Sistemi Complessi, Via Madonna del Piano, 10, 50019 Sesto Fiorentino, Italy; 4 Italian Embassy in Israel, 25 Hamered Street, 68125 Tel Aviv, Israel; 5 CNR-Istituto dei Sistemi Complessi (ISC), via dei Taurini 19, 00185 Roma, Italy; 6 London Institute for Mathematical Sciences, 35a South Street Mayfair, London, W1K 2XF, UK; 7 Linkalab, Complex Systems Computational Laboratory, Cagliari, Italy

## Abstract

Co-evolutionary adaptive mechanisms are not only ubiquitous in nature, but also beneficial for the functioning of a variety of systems. We here consider an adaptive network of oscillators with a stochastic, fitness-based, rule of connectivity, and show that it self-organizes from fragmented and incoherent states to connected and synchronized ones. The synchronization and percolation are associated to abrupt transitions, and they are concurrently (and significantly) enhanced as compared to the non-adaptive case. Finally we provide evidence that only partial adaptation is sufficient to determine these enhancements. Our study, therefore, indicates that inclusion of simple adaptive mechanisms can efficiently describe some emergent features of networked systems’ collective behaviors, and suggests also self-organized ways to control synchronization and percolation in natural and social systems.

Synchronization is possibly the paramount example of how collective behaviors arise in complex systems, as it involves emergence of collective organizations from microscopic interactions of unitary constituents (such as neurons, heart cells, power grids, or crickets^1^). The architecture of such interactions are formally well represented by complex networks^2^,^3^,^4^, and underlying network structure of a system has, indeed, crucial roles in synchronization^5^,^6^. For instance, synchronization on small-world networks can be enhanced compared to regular lattice thanks to the short average distance^7^,^8^ while it could be more difficult on scale-free networks compared to random homogeneous networks due to increased concentration of load to highly connected nodes^9^. Also synchronization can emerges more easily from weighted networks^10^ and scale-free networks and Erdös-Renyi networks follow different paths to synchronization^11^.

The simplest approach to synchronization in networks is assuming a static network structure. However, this approach does not reproduce the behavior observed in real-world systems, where the tendency observed is actually not static, rather dynamic. To cope with this limitation, synchronization have been considered on temporal or time-varying networks^12^,^13^,^14^,^15^. For example, systems of mobile oscillators have been introduced to consider situations where interaction topology changes due to motion of the oscillators^16^,^17^,^18^,^19^. On the other hand, one can observe co-evolution of network structure and network dynamics in many natural and social systems. To take into account these co-evolutionary adaptive mechanisms, various *adaptive* network models were introduced^20^, where structure and the dynamics co-evolve in time^21^,^22^, and states of the nodes shape the structure of their interaction, cooperatively and simultaneously. Synchronization on adaptive networks has been shown interesting phenomena^23^,^24^. Moreover adaptive mechanisms are not only realistic, but they can also enhance and stabilize collective processes^25^,^26^,^27^,^28^, change the order of synchronization^29^, or enable the emergence of meso-scale structures and scale-free properties^30^,^31^.

Current studies on synchronization are, so far, focused on completely percolated networks, i.e., in a situation where all interacting oscillators belong to a single giant connected component. However, real-world systems often show, even temporarily, sparser and non-connected structures, as links between units might well be not *continuously* active^32^,^33^. In such non-connected configurations (where not all nodes belong to a single connected component), achieving global functions (e.g., synchronization) may be hampered by the absence of stable interactions between the units.

In this paper, we consider an adaptive network of oscillators, where every unit (i.e., oscillator) selects its neighborhood on the basis of a homophily principle^34^. Specifically, each oscillator is meant establishing connections with the others that share a similar phase, in analogy to what observed in social and natural systems^34^. It is worth noticing that such a *similarity* might be time-dependent, as distinct oscillators adjust their phases but also (and simultaneously) update the network structure following homophily principles. We will show that our framework qualitatively and quantitatively differs from non-adaptive networks, in that synchronization and percolation transitions come out to be substantially enhanced.

## The Adaptive Network Model

We start by considering a network of *N* (Kuramoto-type) phase oscillators^35^,^36^, whose time evolution is ruled by:


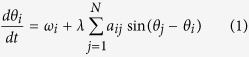


where *ω*_*i*_ (*θ*_*i*_) is the natural frequency (the instantaneous phase) of oscillator *i* drawn from a uniform distribution in the range [−1, 1], *λ* is the coupling strength, and {*a*_*ij*_} are the elements of the network’s adjacency matrix.

The structure of connections is given by the so-called *fitness* or *hidden variable* network model^37^,^38^, which is a generalized Erdös-Reyni (ER) model. The distinctive character of such a model is that the topology is fully shaped by the fitness of the nodes (herein associated to the oscillators’ phases) while the topology is given by a constant probability in the ER model. Accordingly the connection probability between two node *i* and *j* at time *t* is determined by a given function *f*(*θ*_*i*_, *θ*_*j*_). While the form of function *f* can be, in general, arbitrary, we here consider it to follow a homophily principle, through which oscillators with more similar phases are more likely to be connected. For the sake of exemplification, we then define the function *f* as follows:


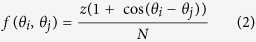


where *z* is a positive parameter, *f*(*θ*_*i*_, *θ*_*j*_) = 2*z*/*N* if *θ*_*i*_ = *θ*_*j*_ and *f*(*θ*_*i*_, *θ*_*j*_) = 0 if |*θ*_*i*_ − *θ*_*j*_| = *π*. If two oscillators feature close enough phases (i.e., |*θ*_*i*_ − *θ*_*j*_| ~ 0), they are then more likely to establish a link, with probability 2*z*/*N*. The expectation is therefore that higher *z* values would lead to more connected network structure, while higher *λ* values would result into more coherent dynamical state. We assume that at each time step the phases of oscillators are updated by Eq. 1 and at the same time step, with a coupling probability *P*, the network topology is shaped by Eq. 2. In this study, without specific indication, we consider the case of *P* = 1.0. For comparison we show the results with *P* = 0.5 and *P* = 0.2, which are very similar with the case of *P* = 1.0, in the Supplementary Information.

## Results

In our simulations, performed with a 4th order Runge-Kutta method and a time-step Δ*t* = 0.02 (See the Supplementary Information for the case of Δ*t* = 0.05 and Δ*t* = 0.1 for comparison), we consider a network size *N* = 300 (See the Supplementary Information for cases of *N* = 150 and *N* = 600). We assign initial conditions for the oscillators’ phases from a uniformly distributed distribution in the range [−*π, π*], while the initial network structure is taken to be that extracted from Eq. (2) with the given initial phases. At each time step of the integration, oscillators’ phases evolve by Eq. (1), and (simultaneously) network structure is reshaped by Eq. (2). To compare with, the non-adaptive evolution is also simulated, where the structure of the network is determined by Eq. (2) *only initially*.

The degree of synchronization can be monitored by the synchronization order parameter:


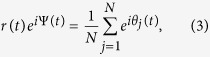


whose modulus (*r*(*t*) ∈ [0, 1]) measures actually the system’s phase coherence (*r* = 1 for the fully synchronized regime, *r* ~ 0 for the incoherent state). Ψ(*t*) is instead the average phase of the system. For percolation, we consider the relative size of the largest connected component *s*(*t*) as the order parameter. For each parameter *r*(*t*) and *s*(*t*), we furthermore define *R* and *S* as the respective steady state values, i.e. the values obtained by averaging over 500 steps, and after 3,000 transient steps.

**Figure 1:**
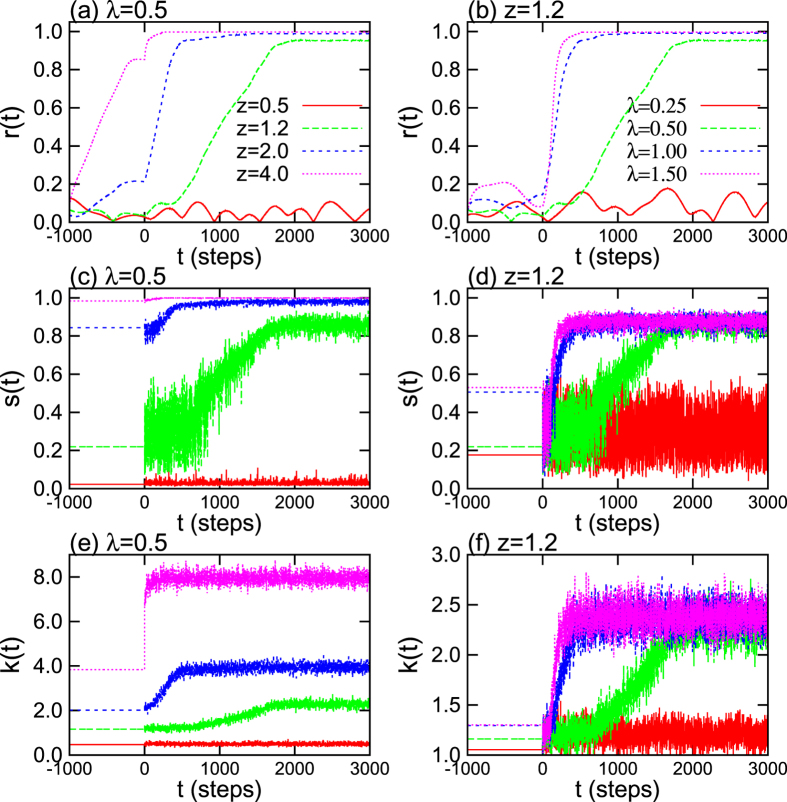
Time evolution of *r*(*t*) (**a,b**), *s*(*t*) (**c,d**) and of the network’s average degree *k*(*t*) (**e,f**). (**a,c,e**) *λ* = 0.5; (**b,d,f**) *z* = 1.2. Color codes in the legends of (**a,b**).

Figure 1 reports the time evolution of *r*(*t*) and *s*(*t*), at different values of the control parameters *z* and *λ*. When *t* < 0, the time evolution of the order parameters is determined by the fixed network structure constructed by Eq. 2 with the initial phases (i.e., non-adaptive networks), whereas the network structure (starting from *t* = 0) is updated by Eq. 2 at every time step. In Fig. 1(a,c), *r*(*t*) and *s*(*t*) are plotted at *λ* = 0.5 and varying *z*, respectively while Fig. 1(b,d) reports *r*(*t*) and *s*(*t*) (at fixed *z* = 1.2) by varying *λ*. A clear enhancement of synchronization and percolation is simultaneously observed for most values of *λ* and *z* (except when *z* = 0.5 and *λ* = 0.5, or when *z* = 1.2 and *λ* = 0.25). The evolution of the network’s average degree *k*(*t*) [Fig. 1(e,f)] reveals that adaptation leads actually to an increase of the average degree, which may explain the concurrent enhancement of percolation and synchronization in the adaptive network.

**Figure 2:**
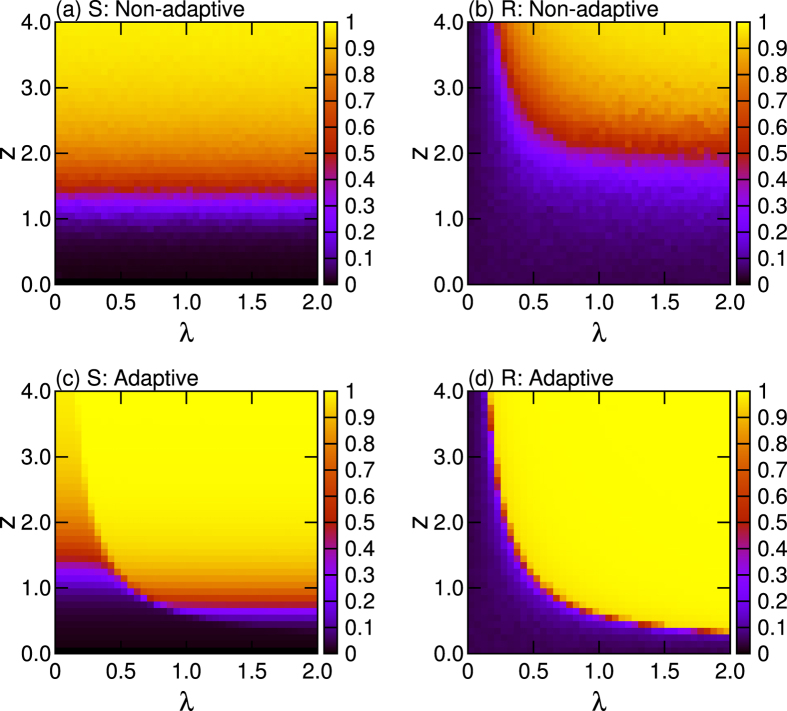
Phase diagrams of the non adaptive (**a,b**) and adaptive (**c,d**) models. Panels refer to the percolation indicator *S* (**a,c**) and the synchronization indicator *R* (**b,d**). For each *z* and *λ*, data refer to ensemble averages over 40 different realizations.

Figure 2 accounts for *S* and *R* in the parameter space (*λ, z*). The percolation transition in the non-adaptive network only depends on *z* [as shown in Fig. 2(a)]. We observe existence of typical percolation transitions within the subcritical regime (*S* ~ 0.0) of *z* < 1.0, the critical regime of *z* ~ 1.0, and the supercritical regime (0.0 < *S* < 1.0) of 1.0 < *z* < 3.0, and also the connected regime (*S* ~ 1.0) is observed for *z* > 3.0. As shown in Fig. 2(b), synchronization in the non-adaptive case depends on the specific percolation state the network is attaining. Fully incoherent states (*R* < 0.05) are observed in sub-critical and critical regime (*z* < 1.0) regardless of *λ*. Partial synchronization (0.1 < *R* < 0.9) is observed, instead, in supercritical regimes, and highly synchronized states emerge only in the connected regime (*z* > 3.0).

On the other hand, significant enhancement of percolation and synchronization is evident in Fig. 2(c,d). In particular, the enhancement is substantial in the region of *z* < 3.0 corresponding to the non-connected regimes in the non-adaptive network. In particular, the percolation indicator *S* depends not only on *z*, but also on *λ*, and (when *λ* increases) the giant connected component emerges even for smaller values of *z*.

Furthermore, synchronization is actually boosted in the adaptive network [Fig. 2(d)]. Similarly to percolation, the enhancement is here predominant in low connection ability regions (*z* < 3.0). Interestingly enough, also some not-fully connected regions (*S* < 1.0) still can display highly coherent states (*R* ~ 1). The conclusions that can be drawn from our results is that the adaptive mechanism actually creates a positive feedback loop between network’s structure and dynamics, thence supporting the ubiquity of synchronized and connected components in complex systems under limited resources for interactions.

**Figure 3:**
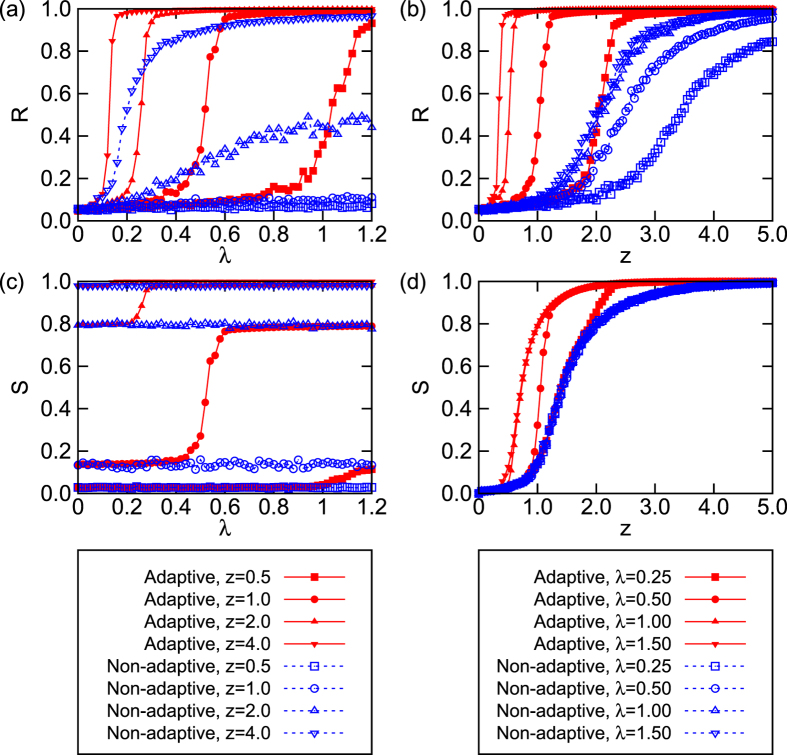
*R* (**a,b**) and *S* (**c,d**) for adaptive and non-adaptive networks. (**a**) *R vs. λ* at different *z* values; (**b**) *R vs. z* at different *λ* values; (**c**) *S vs. λ* at different *z* values; (**d**) *S vs. z* at different *λ* values. Legends (in the bottom panels) have to be referred to for the understanding of the used parameters’ values. Data refer to ensemble averages over 40 realizations.

The adaptive mechanisms here considered not only enhance synchronization and percolation, but also make both transitions more abrupt. In other words both transitions in the adaptive networks are more sensitive to the coupling strength *λ* and to the connectivity parameter *z* than the transitions in the non-adaptive networks. Note that, in this sense, here we do not consider the observed transitions as so-called explosive synchronization^39^ or percolation^40^. In Fig. 3 we report *R* [panels (a) and (b)] and *S* [panels (c) and (d)] as a function of *λ* at fixed *z*, as well as varying *z* at fixed *λ*. For non-adaptive networks, the passage from incoherent to coherent states (and that from fragmented to percolated structures) features typical traits of second-order transitions, while adaptive networks displays abrupt patterns. The case of percolation transition shows, actually, more interesting patterns. When *z* is fixed, *S* in the non-adaptive network does not depend on *λ* [as shown in Figs 2(a) and 3(c)]. However, *S* in the adaptive case shows a clear percolation transition with growing *λ* when *z* < 4.0 [see the red lines with filled symbols in Fig. 3(c)]. Interestingly, there is no difference in the behavior of *S* (before the transition) between the adaptive and non-adaptive case. Only above certain values of *λ*, the percolation transition assumes a characteristic “first-order-type nature” [as seen in Fig. 3(d)]. It is notable that, although the interplay between network evolution and dynamics happens here simultaneously, the transition to synchrony seems to occur at lower *z* or *λ* values, actually, than the percolation transition.

**Figure 4:**
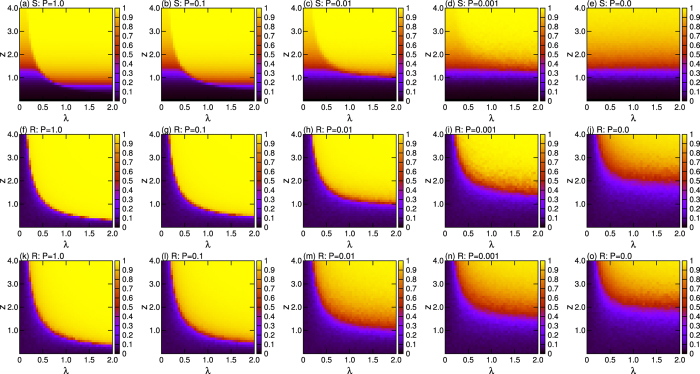
*S* (top row) and *R* (middle row) in the parameter space (*z, λ*) for the adaptive network with different coupling probability *P*. Bottom row reports, instead, *R* (in the same parameter space) for blinking networks with different coupling probability *P*. Once again, data refer to ensemble average over 40 different realizations for each *z* and *λ*.

While the effect of the interplay between topological and dynamical evolution of nodes appears to be clear, it is of the highest importance orienting the study to the inspection of the timescales at which the two phenomena appear. In particular, if updating network structure costs more than updating states of oscillators, it is necessary to check whether adaptive mechanisms should be applied at every time step or, instead, just few applications of them are actually sufficient to determine the observed enhancements. The issue is here addressed by introducing a coupling probability *P* between dynamics of oscillators and structural evolution, namely by updating the network structure [via Eq. (2)] with probability *P* at each time step. The limit *P* = 0 recovers a non-adaptive network model, while *P* = 1.0 corresponds to a totally adaptive case. In Fig. 4 we report *S* (top row) and *R* (middle row) from the cases of *P* = 1, 0.1, 0.01, 0.001 and 0. Remarkably, one observes that both transitions (to percolation and synchrony) are significantly enhanced along all the finite range of *P*, including *P* = 0.001. This fact has significant implications, in the sense that one can actually intervene on the collective behaviors of a given system, only with a few applications of our proposed adaptive mechanism.

It was recently reported that blinking networks (i.e. topologies of interactions which change over timescale much faster than that of the network units’ dynamics), can actually enhance synchronization^41^,^42^. As our adaptive model also can have such a ‘blinking’ nature (when *P* ~ 1.0), it is therefore mandatory to comparatively investigate on how much the observed enhancement in synchronization has a route within the yet known blinking effects. To this purpose, we consider a blinking network of oscillators (which is exactly the same as the considered adaptive network) with a topology updated by a random probability *Q*, and which gives the same number of links at the initial step given by Eq. 2. Note that whether updating topology or not at each time step depends on the coupling probability *P* in both of the adaptive network and the blinking network while the connections between the oscillators are given by Eq. 2 in the adaptive network but by the random probability *Q* in the blinking network. The bottom panels of Fig. 4 reports the values of *R* for such a latter, blinking, network as function of *λ* and *z*, with varying *P*. When *P* = 1.0, one notices that the blinking effect is, indeed, quite strong. However, the effect vanishes rapidly with decreasing *P*. This indicates that our adaptive mechanism may enhance synchronization *only partially* due to blinking effects, whereas significant other contributions exist. It is also noticeable that no enhancement in percolation exists at all in the blinking framework, due punctually to the lack of feedback between dynamics of oscillators and topological evolution.

## Discussion

In conclusion, complex networks need to stay in connected and synchronized states, in order to perform integrated and coherent functions. However, when the units have only limited ability to connect to each other, it is of paramount importance understanding how the networks self-organize from fragmented and incoherent states to connected and synchronized states. We have considered an adaptive model, where connections between nodes are ruled by a positive feedback loop connecting structural evolution (driven by a fitness model) and nodal dynamics (driven by the Kuramoto model). We actually gave evidence that such an adaptive framework enhances substantially synchronization and percolation, while non-adaptive counterparts fail to reach synchronization and percolation in the non-connected regime. This indicates that co-evolutionary adaptive networks are not only more realistic descriptions of complex systems, but also they are beneficial for the correct and robust functioning of complex systems.

The observed enhancement of synchronization and percolation shed actually light on how one can control such two processes in a spontaneous, or self-organized, way^22^. In particular, as shown in our Fig. 4, the needed coupling has not to be very strong, thus suggesting that the control of unwanted events emerging through synchronization (such as epileptic seizure or market crashes) could be easily achieved by just (properly) coupling or decoupling network’s structure evolution and dynamics. In this sense, our findings suggest efficient control methods to maintain an integrated functioning of natural and social systems.

## Additional Information

**How to cite this article**: Eom, Y.-H. *et al*. Concurrent enhancement of percolation and synchronization in adaptive networks. *Sci. Rep.* **6**, 27111; doi: 10.1038/srep27111 (2016).

## Supplementary Material

Supplementary Information
