# Supplementary material for: Concurrent enhancement of percolation and synchronization in adaptive networks
Source: Sci Rep. 2016 Jun 2;6:27111. doi: 10.1038/srep27111 (PMC4890019; doi:10.1038/srep27111)
Supplement: Supplementary Information [file srep27111-s1.pdf]

# Concurrent enhancement of percolation and synchronization in adaptive networks (Supplementary Information)

Young-Ho Eom[1,2], Stefano Boccaletti[3,4], Guido Caldarelli[1,5,6,7]
[1] *IMT School for Advanced Studies Lucca, Piazza San Francesco 19, Lucca 55100, Italy*
[2] *Departamento de Matemáticas, Universidad Carlos III de Madrid, 28911 Leganés, Spain*
[3] *CNR-Istituto dei Sistemi Complessi, Via Madonna del Piano, 10, 50019 Sesto Fiorentino, Italy*
[4] *Italian Embassy in Israel, 25 Hamered Street, 68125 Tel Aviv, Israel*
[5] *Istituto dei Sistemi Complessi (ISC), via dei Taurini 19, 00185 Roma, Italy*
[6] *London Institute for Mathematical Sciences, 35a South Street Mayfair, London, W1K 2XF, UK*
[7] *Linkalab, Complex Systems Computational Laboratory, Cagliari, Italy*
[*] *Corresponding author: GC (guido.caldarelli@imtlucca.it)*

# I. CASES OF DIFFERENT TIME SCALE BETWEEN OSCILLATORS' DYNAMICS AND STRUCTURAL EVOLUTION

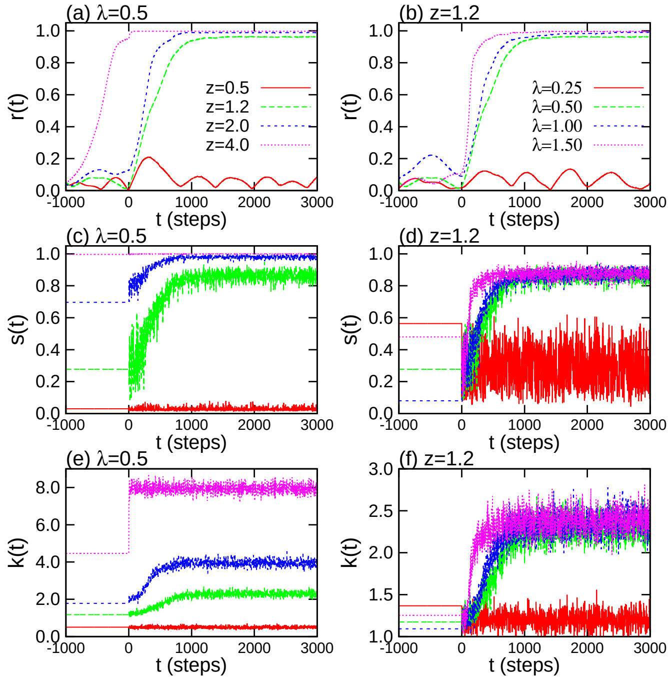


FIG. 1. Time evolution of $r(t)$ (a-b), $s(t)$ (c-d) and of the network's average degree $k(t)$ (e-f) for $P = 0.5$ and $N = 300$. (a, c, and e) $\lambda = 0.5$; (b, d, and f) $z = 1.2$. Color codes in the legends of (a) and (b).

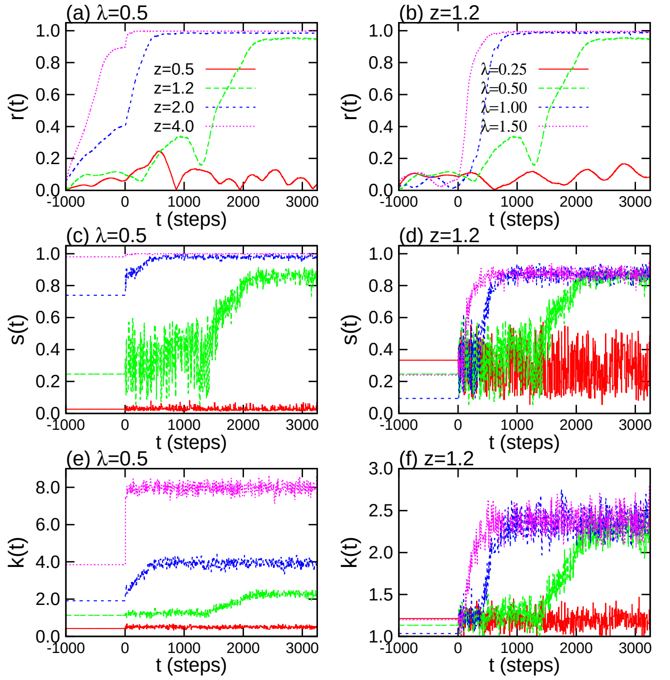


FIG. 2. Time evolution of $r(t)$ (a-b), $s(t)$ (c-d) and of the network's average degree $k(t)$ (e-f) for $P = 0.2$ and $N = 300$. (a, c, and e) $\lambda = 0.5$; (b, d, and f) $z = 1.2$. Color codes in the legends of (a) and (b).

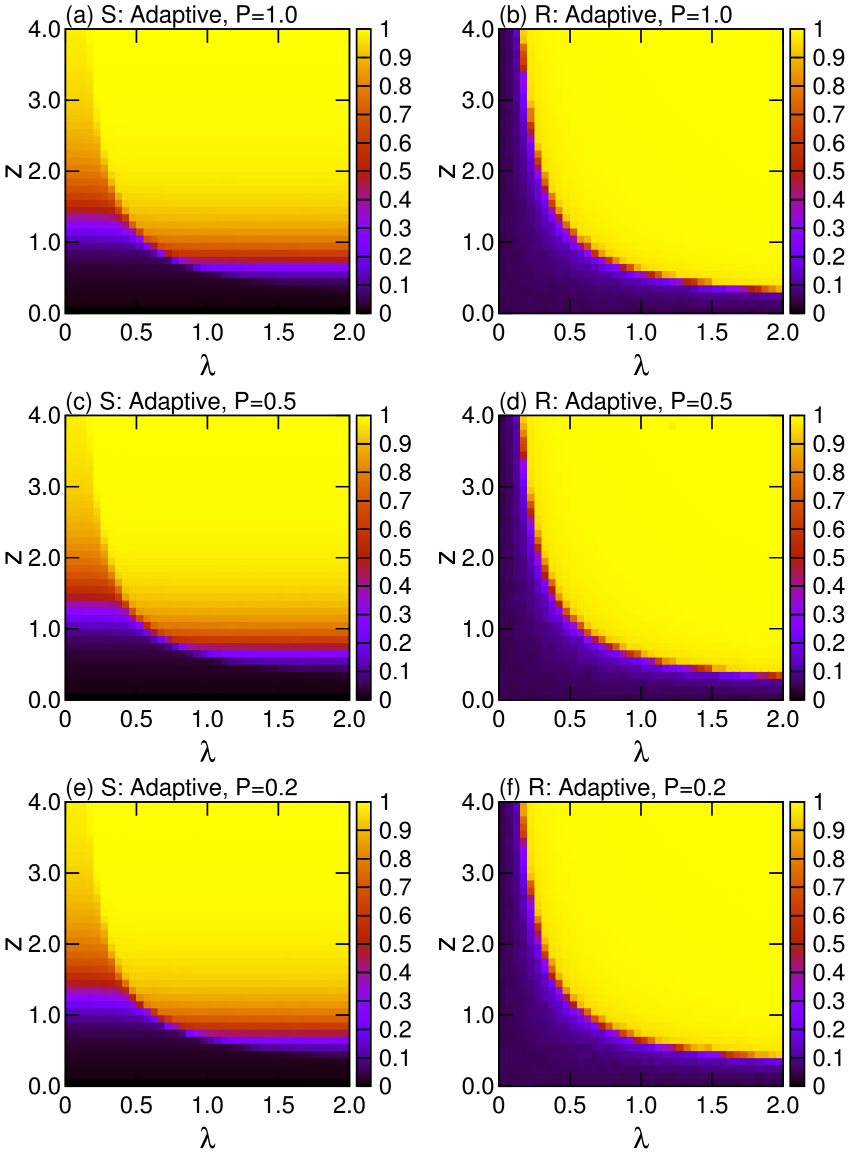


FIG. 3. Phase diagrams of adaptive models with $N = 300$ and varying $P$ ($P = 1.0$, 0.5, and 0.2). Panels refer to the percolation indicator $S$ (a,c,e) and the synchronization indicator $R$ (b,d,f). For each $z$ and $\lambda$, data refer to ensemble averages over 40 different realizations.

## II. CASES OF DIFFERENT SYSTEM SIZES

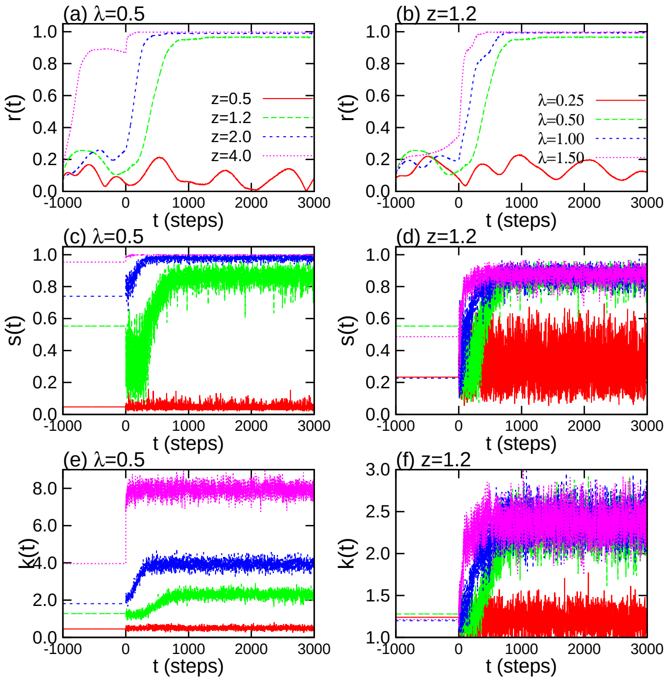


FIG. 4. Time evolution of $r(t)$ (a-b), $s(t)$ (c-d) and of the network's average degree $k(t)$ (e-f) for $P = 1$ and $N = 150$. (a, c, and e) $\lambda = 0.5$; (b, d, and f) $z = 1.2$. Color codes in the legends of (a) and (b).

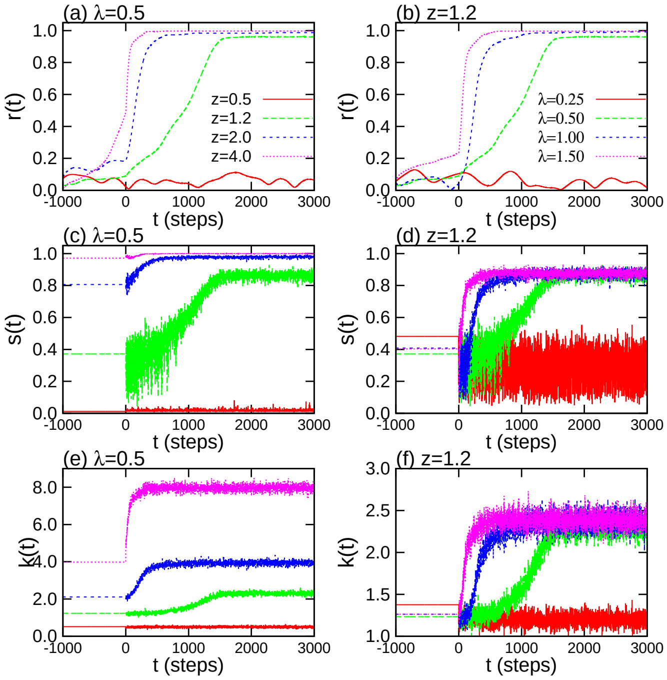


FIG. 5. Time evolution of $r(t)$ (a-b), $s(t)$ (c-d) and of the network's average degree $k(t)$ (e-f) for $P = 1$ and $N = 600$. (a, c, and e) $\lambda = 0.5$; (b, d, and f) $z = 1.2$. Color codes in the legends of (a) and (b).

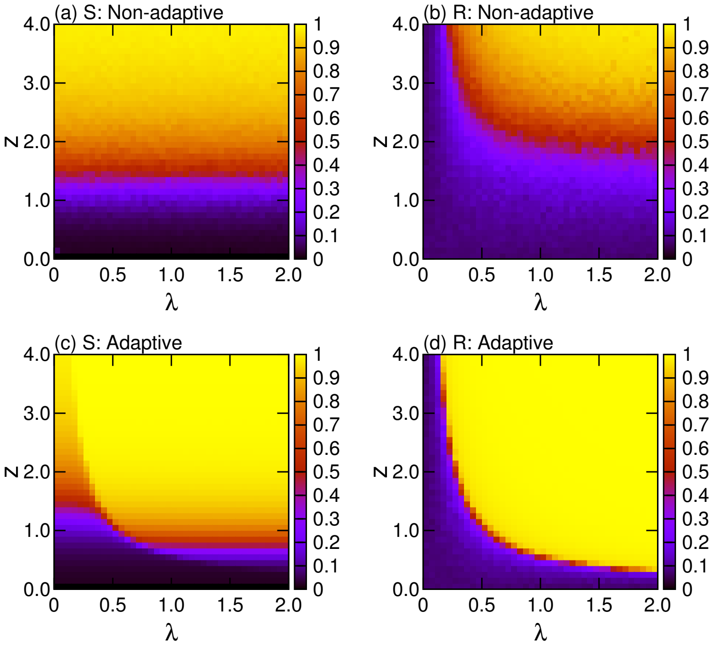


FIG. 6. Phase diagrams of the non adaptive (a,b) and adaptive(c,d) models with $N = 150$ and $P = 1$. Panels refer to the percolation indicator $S$ (a,c) and the synchronization indicator $R$ (b,d). For each $z$ and $\lambda$, data refer to ensemble averages over 40 different realizations.

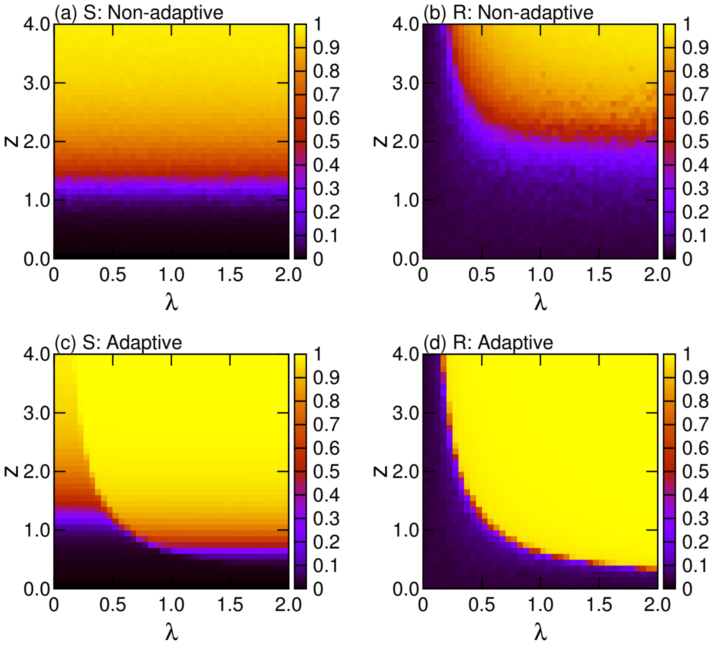


FIG. 7. Phase diagrams of the non adaptive (a,b) and adaptive(c,d) models with $N = 600$ and $P = 1$. Panels refer to the percolation indicator $S$ (a,c) and the synchronization indicator $R$ (b,d). For each $z$ and $\lambda$, data refer to ensemble averages over 20 different realizations.

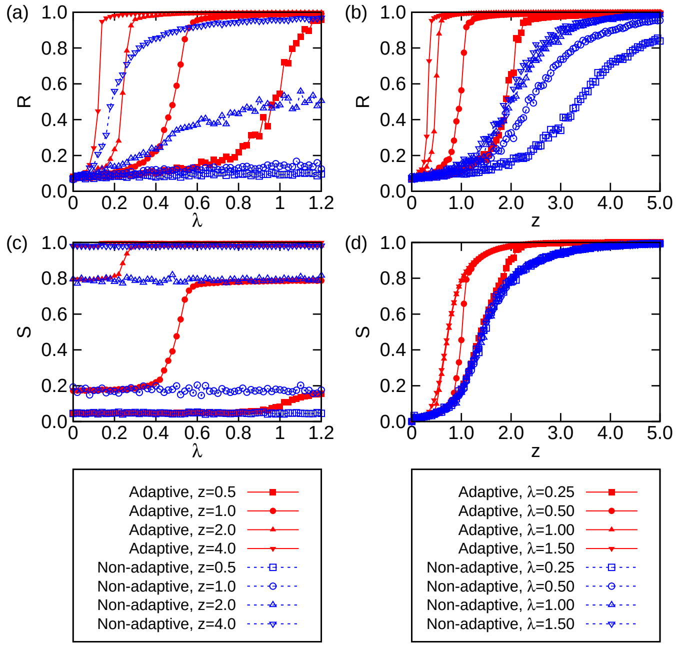


FIG. 8. $R$ (a,b) and $S$ (c,d) for adaptive and non-adaptive networks with $N = 150$ and $P = 1.0$. (a) $R$ *vs.* $\lambda$ at different $z$ values; (b) $R$ *vs.* $z$ at different $\lambda$ values; (c) $S$ *vs.* $\lambda$ at different $z$ values; (d) $S$ *vs.* $z$ at different $\lambda$ values. Legends (in the bottom panels) have to be referred to for the understanding of the used parameters' values. Data refer to ensemble averages over 40 realizations.

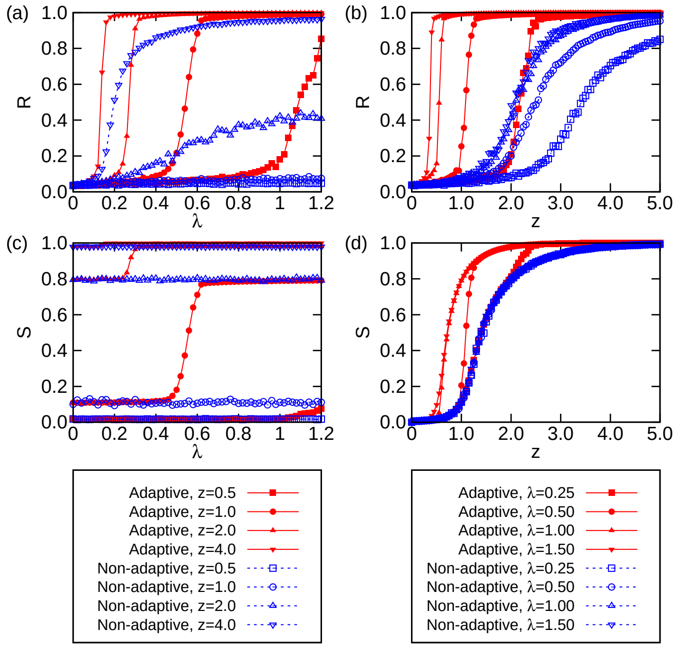


FIG. 9. $R$ (a,b) and $S$ (c,d) for adaptive and non-adaptive networks with $N = 600$ and $P = 1.0$. (a) $R$ *vs.* $\lambda$ at different $z$ values; (b) $R$ *vs.* $z$ at different $\lambda$ values; (c) $S$ *vs.* $\lambda$ at different $z$ values; (d) $S$ *vs.* $z$ at different $\lambda$ values. Legends (in the bottom panels) have to be referred to for the understanding of the used parameters' values. Data refer to ensemble averages over 40 realizations.

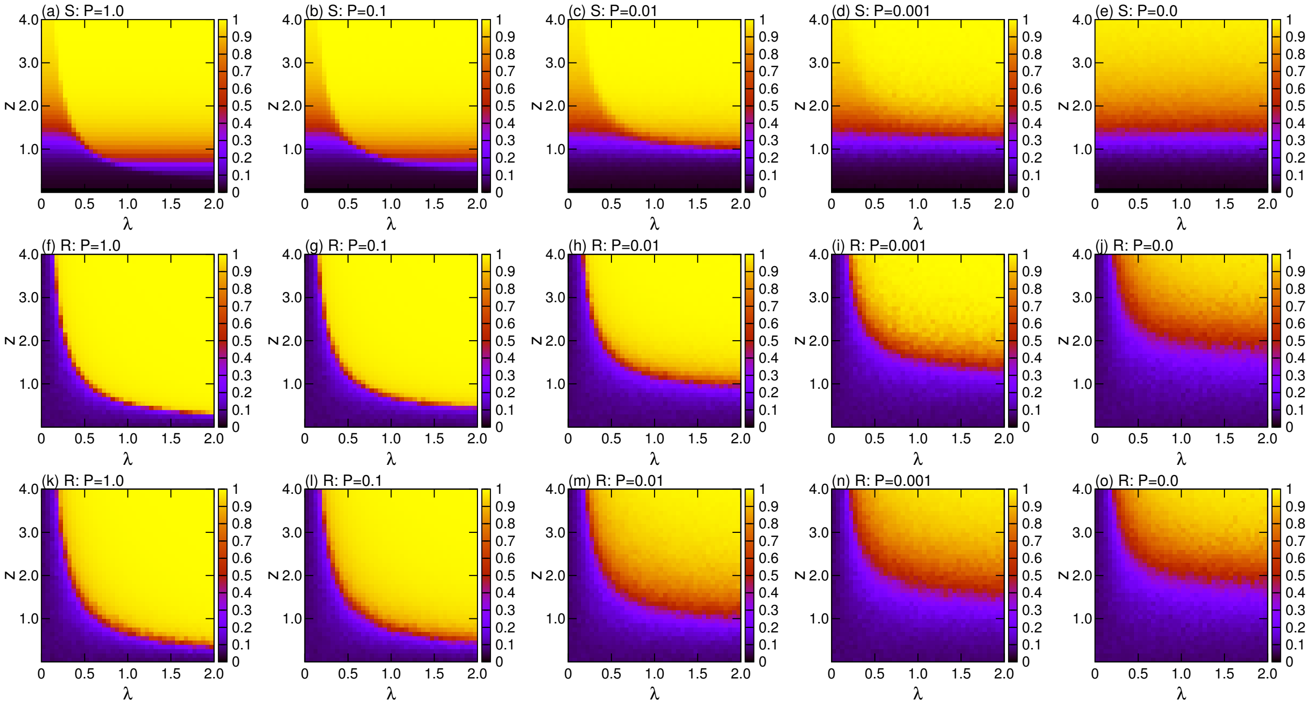


FIG. 10. $S$ (top row) and $R$ (middle row) in the parameter space $(z, \lambda)$ for the adaptive network of size $N = 150$ with different coupling probability $P$. Bottom row reports, instead, $R$ (in the same parameter space) for blinking networks with different coupling probability $P$. Once again, data refer to ensemble average over 40 different realizations for each $z$ and $\lambda$.

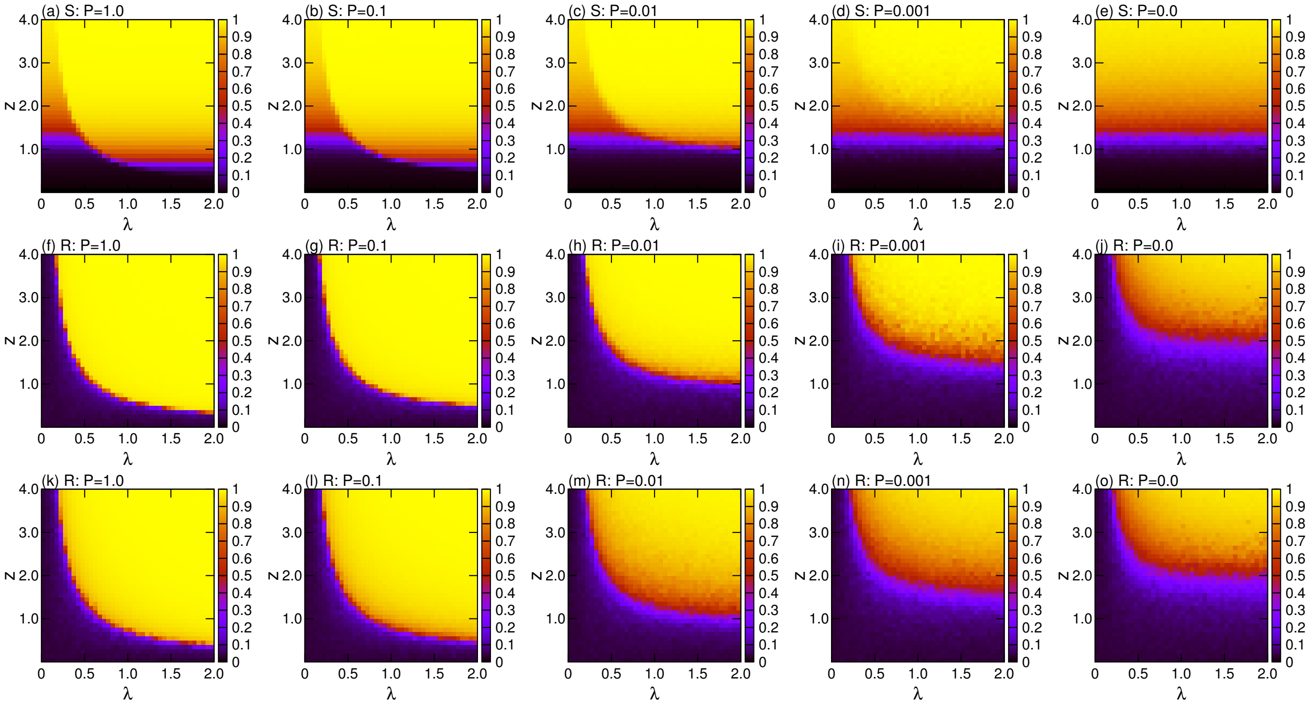


FIG. 11. $S$ (top row) and $R$ (middle row) in the parameter space $(z, \lambda)$ for the adaptive network of size $N = 600$ with different coupling probability $P$. Bottom row reports, instead, $R$ (in the same parameter space) for blinking networks with different coupling probability $P$. Once again, data refer to ensemble average over 20 different realizations for each $z$ and $\lambda$.

## III. CASES OF DIFFERENT STEP-SIZES IN RUNGE-KUTTA METHOD

So far, all the results in the main manuscript and in the supplementary information are obtained from Runge-Kutta method with step-size $\Delta t = 0.02$. We also consider the cases of different step-sizes of $\Delta t = 0.05$ and 0.1 for comparison.

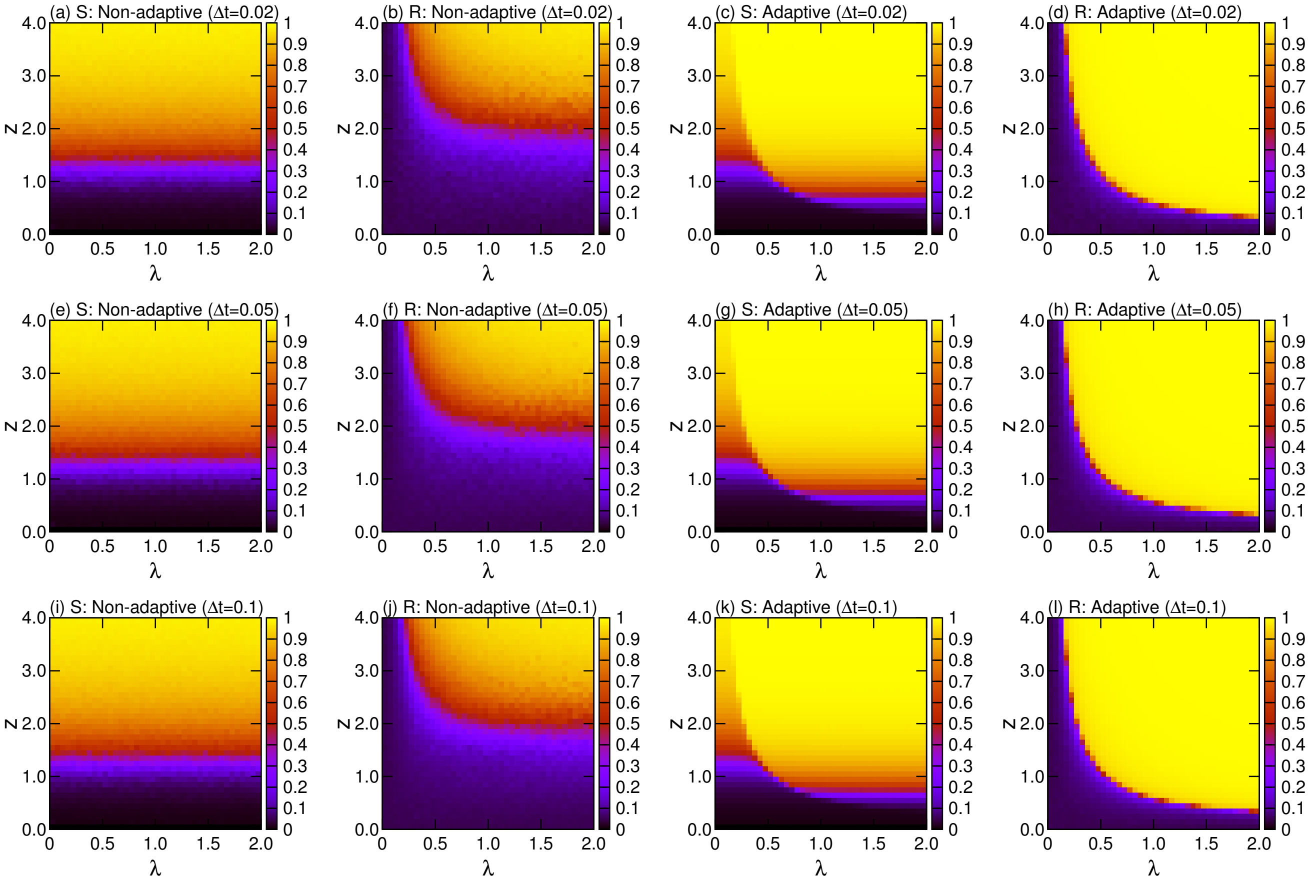


FIG. 12. Phase diagrams of the non-adaptive (a,b,e,f,i,j) and adaptive(c,d,g,h,k,l) models. Panels refer to the percolation indicator S (a,c,e,g,i,k) and the synchronization indicator R (b,d,f,h,j,l). Panels from (a) to (d) are from simulations with $\Delta t = 0.02$. Panels from (e) to (h) are from simulations with $\Delta t = 0.05$. Panels from (i) to (l) are from simulations with $\Delta t = 0.10$. For each z and l, data refer to ensemble averages over 40 different realizations
